# Conceptualising characteristics of resources withdrawal from medical services: a systematic qualitative synthesis

**DOI:** 10.1186/s12961-020-00630-9

**Published:** 2020-10-28

**Authors:** Mark Embrett, Glen E. Randall, John N. Lavis, Michelle L. Dion

**Affiliations:** 1 Faculty of Health, School of Nursing, Dalhouise University, 5869 University Avenue, PO BOX 15000, Halifax, Nova Scotia B3H 4R2 Canada; 2 St. Francis Xavier University, 4130 University Avenue, Antigonish, Nova Scotia B2G2W5 Canada; 3 Health Policy and Management, DeGroote School of Business, McMaster University, Hamilton, Ontario L8S4M4 Canada; 4 McMaster University, DSB-229, 1280 Main Street West, Hamilton, Ontario L8S 4M4 Canada; 5 Department of Health Research Methods, Evidence, and Impact, McMaster University, Hamilton, Ontario L8S4L6 Canada; 6 McMaster Health Forum, MML-417, 1280 Main St. West, Hamilton, Ontario L8S4L6 Canada; 7 Department of Political Science, McMaster University, Hamilton, Ontario L8S4L6 Canada; 8 Kenneth Taylor Hall (KTH) 533, 1280 Main St. West, Hamilton, Ontario L8S4L6 Canada

**Keywords:** Disinvestment, Rationing, Qualitative synthesis, Resource withdrawal, Priority-setting

## Abstract

**Background:**

Terms used to describe government-led resource withdrawal from ineffective and unsafe medical services, including ‘rationing’ and ‘disinvestment’, have tended to be used interchangeably, despite having distinct characteristics. This lack of descriptive precision for arguably distinct terms contributes to the obscurity that hinders effective communication and the achievement of evidence-based decision-making. The objectives of this study are to (1) identify the various terms used to describe resource withdrawal and (2) propose definitions for the key or foundational terms, which includes a clear description of the unique characteristics of each.

**Methods:**

This is a systematic qualitative synthesis of characteristics and terms found through a search of the academic and grey literature. This approach involved identifying commonly used resource withdrawal terms, extracting data about resource withdrawal characteristics associated with each term and conducting a comparative analysis by categorising elements as antecedents, attributes or outcomes.

**Results:**

Findings from an analysis of 106 documents demonstrated that terms used to describe resource withdrawal are inconsistently defined and applied. The characteristics associated with these terms, mainly antecedents and attributes, are used interchangeably by many authors but are differentiated by others. Our analysis resulted in the development of a framework that organises these characteristics to demonstrate the unique attributes associated with each term. To enhance precision, these terms were classified as either policy options or patient health outcomes and refined definitions for rationing and disinvestment were developed. Rationing was defined as resource withdrawal that denies, on average, patient health benefits. Disinvestment was defined as resource withdrawal that results in, on average, improved or no change in health benefits.

**Conclusion:**

Agreement on the definition of various resource withdrawal terms and their key characteristics is required for transparent government decision-making regarding medical service withdrawal. This systematic qualitative synthesis presents the proposed definitions of resource withdrawal terms that will promote consistency, benefit public policy dialogue and enhance the policy-making process for health systems.

## Background

Many governments have expressed significant concern over the rising costs of healthcare [1, 2]. Public spending on unsafe, ineffective and inefficient medical services continues to contribute to rising healthcare costs and minimises the benefits realised by health systems [1]. Direct government intervention through the modification of publicly funded health insurance coverage is one potential avenue for limiting inappropriate care. Researchers and health professionals have recommended mechanisms for discontinuing funding (or facilitating resource withdrawal); however, the complexities associated with these processes are not well understood, particularly in light of the confusing and inconsistent terminology associated with resource withdrawal [2–6]. Terms used to describe government-led resource withdrawal from ineffective and unsafe medical services, including ‘rationing’ and ‘disinvestment’, tend to be used interchangeably, despite their distinct characteristics. This lack of descriptive precision for distinct terms contributes to a lack of clarity that hinders effective communication and achieving evidence-based decision-making. For this reason, clarification of terminology is necessary to describe the situations in which resource withdrawal occurs, to understand which factors influence resource withdrawal outcomes, and to communicate resource withdrawal information accurately [7].

During a time of unparalleled health expenditure, there exists a widespread belief that many health systems achieve a poor return on investment. Empirical evidence, primarily from high-income countries, indicates that only 20% of insured medical services have well-supported evidence regarding treatment effectiveness [8], only 40% of patients receive treatments with proven effectiveness, and as much as 25% of patients receive treatments that are unnecessary or harmful [9]. In low- and middle-income countries, where there is little transparency or use of existing health outcome measures, up to 15% of overall deaths (8.4 million) are attributed to poor quality care [10]. These findings suggest that there are many instances of patients receiving inappropriate care [11] and that resources are going to waste. Medical care may be considered appropriate if, on average, the probability of benefits of providing a service sufficiently exceed the probability of harm [12].

As health technology assessment (HTA) becomes routinised in many high-income countries, such as Canada, the United States, the United Kingdom, Australia, Belgium, Germany and Sweden (among others) [13], and in some middle-income countries [14], researchers and healthcare organisations have recommended that governments embrace an explicit, evidence-based, routinised process for resource withdrawal from inappropriate services [15]. Proponents of evidence-based medicine (EBM), cost-effectiveness research (CER) and HTA have echoed these recommendations [8, 16–19]. Broadly speaking, resource withdrawal refers to the reduction, restriction or removal of public entitlement to insured medical services (drugs, devices, diagnostics and surgical procedures).

Through recent systematic reviews [19], geographical service utilisation analyses [20], health technology reassessment (HTR) [16] and other studies [21], several proposals on the most effective way to identify inappropriate services have been made. Despite calls for action, resource withdrawal from inappropriate services using such an explicit, evidence-based approach has not yet become routine within most countries. A lack of these formal mechanisms results in the continuation of funding for inappropriate services that are (1) unsafe or harmful, (2) clinically ineffective, (3) comparatively ineffective (4) and/or cost ineffective [22–26].

Developing routine processes and mechanisms for resource withdrawal remains a challenge for governments for several reasons, including the fact that a large proportion of insured medical services are based on historical practices and experiences rather than on their clinical efficacy and cost effectiveness [8, 27]. There is also difficulty in distinguishing between different healthcare services to remedy an ailment, categorising and organising particular healthcare services for priority-setting, and assessing services in a way that is both evidence based and socially fair [28].

A government’s reluctance towards developing a process for withdrawing resources is often influenced by the political implications associated with the explicit removal of resources from health services. Contributing to the political difficulty is the absence of well-defined resource withdrawal terms, a void which leads to heightened levels of confusion among medical professionals, policy-makers, health organisations, politicians, patients/taxpayers and other stakeholders [3–5, 18, 29]. Heightened confusion may in turn lead to disagreement regarding various aspects of government policies that target resource withdrawal, which contributes to the resistance towards developing explicit, routinised processes.

### Objectives: clarifying concepts

Before advancing a common understanding of how governments can design a regime for evidence-based resource withdrawal, the definitions of important terminology must be clarified. Unclear definitions make analysing the value and implications of subject-specific terms difficult [30, 31]. When unclear definitions exist, further analysis is needed to provide clarity and enhance communication regarding the specific topic area, which in this case is government-led resource withdrawal from medical services. To this end, the objectives of the present study were to (1) identify the various terms used to describe resource withdrawal and (2) propose definitions for the key or foundational terms, which includes a clear description of the unique characteristics (attributes, antecedents and outcomes) of each.

## Methods

### Procedure of systematic qualitative synthesis

This study conducted a systematic qualitative synthesis, described by Saini and Shlonsky [32], to collect and analyse the use of resource withdrawal characteristics and terms within academic and grey literature. The synthesis consists of 11 steps, the first of which was setting objectives (described above). This approach to qualitative synthesis methodology is intended to enhance understanding of how different concepts connect and interact with one another. Under this methodology, the lead researcher’s (ME) task was to utilise secondary analysis of the existing literature as data on the subject (resource withdrawal from medical services) to investigate the relationships between terms and define important terms using their characteristics, provided that sufficient data exist. The result of the synthesis was the researcher’s interpretation of the findings of the original eligible studies [33]. In this way, the primary comparative data for the present analysis was the description and interpretation of the use of resource withdrawal terms’ characteristics in the academic and grey literature.

To define the characteristics of each term we adopted Rodger’s approach to concept analysis, which emphasises the description and clarification of terms to further understand their meaning and conduct research into the phenomena/ideas they represent [7, 34]. The primary characteristic concepts include the antecedents, attributes and outcomes. Each were defined in the following manner [7]:

1. An antecedent was an event that was reported to have logically preceded the resource withdrawal.
2. An attribute was an inherent quality or feature of the resource withdrawal process.
3. An outcome was an occurrence that results from resource withdrawal at the level of services (e.g. reduced, restricted or denied), patients (e.g. improved, maintained or reduced health outcome) or the health system (e.g. efficiency, spending).

Analysis oriented around these characteristics, similar to a concept analysis approach, is often used in nursing research [35–37]. Patterns of characteristics’ use that emerged in the analysis were used to help synthesise characteristics and meanings of specific terms were identified as important through an iterative analysis of the literature.

#### Eligibility/scope of research

The second step was to establish the breadth of the research. Given that the review focused on government-led resource withdrawal from medical services, we included articles that used resource withdrawal terms related to government regulation of resources used for medical services. Articles and policy documents were included if they (1) described/interpreted a resource withdrawal activity (e.g. delisting, rationing, disinvesting, decommissioning) that was related to medical service(s) (drugs, devices, diagnostics and surgical procedures); (2) described/interpreted the role of government (national/subnational/local) in the process of resource withdrawal; (3) focused on one or more of the Organisation of Economic Cooperation and Development (OECD) countries; and (4) was published in English. OECD countries were chosen in order to focus on countries with health systems in which government have a well-developed role in regulating and funding medical services.

#### Information sources

Step three included undertaking searches and information retrieval. Academic electronic databases provided the platform for searching scholarly papers. The grey literature included government policy reports and non-governmental organisation reports. The grey literature was obtained through websites that were originally identified using the Canadian Agency for Drugs and Technology in Health advice for searching health-related grey literature [13]. Specific articles were also identified through reference chaining and the provision of alerts from electronic databases advising that newly published articles matched search criteria.

Relevant studies were identified through a search of the following eight electronic databases and platforms: CINAHL, Embase, HealthSTAR, Medline, ProQuest, PsycINFO, Scholar Portal and Web of Science. The search was conducted in November 2014. Keywords related to resource withdrawal (“disinvest” OR “divest” OR “decommission” OR “delist” OR “ration” OR “deinsure” OR “displace” OR “replace” OR “retract” OR “restrict”) and health service type (“health service” OR “healthcare service” OR “medical service” OR “drug therapy” OR “diagnostic service” OR “laboratory service” OR “technology”) were used. Truncations of terms were searched separately within each database in order to capture all possible iterations of keywords in the literature. The search identified articles that had the listed terms in the title, abstract or keywords. The same set of terms was used to search the grey literature.

#### Data screening extraction

Step four included screening each article’s title and abstract for eligibility. As articles were screened by the primary researcher (ME) and an independent research assistant, eligible articles were classified by study type (step 5). After title and abstract screening, each eligible article’s full text was reviewed to further determine if the paper was eligible (step 6). During full text review, the primary researcher (ME) extracted the following data from all eligible articles: definition, antecedents, attributes, outcomes on service, patient, health system, and medical service type. Characteristics of the studies were also extracted, including study country, objective, policy strategies, findings, methods and implications (step 7). If a term’s definition was explicitly stated, it was copied to the data extraction table. If the articles did not explicitly provide term definition but implied it, the primary investigator developed a definition based on the original study’s implications.

Step eight (quality assessment) was not conducted in this study because a significant majority of the studies were commentaries, essays or opinion pieces with no methods or results to assess. Furthermore, because the objective was to extract definitions of terms and not to report the results of the study, quality assessment was not deemed necessary. After the primary investigator reviewed an initial set of articles at the full text stage, both the primary investigator and a second researcher extracted data from these articles. Results from each researcher’s data extractions were compared for all articles. There were no significant discrepancies to report between the data extractions.

#### Data synthesis

Data comparison and synthesis were conducted iteratively throughout the analytic process by the primary investigator (ME) (step 9). Throughout the extraction of relevant data, information was consistently and thoroughly reviewed and analysed to produce preliminary descriptions of patterns found in the studies. The primary investigator explored relationships both within and among the antecedents, attributes and outcomes as described in the literature. The primary investigator identified and extracted the ways in which characteristics were associated with prominent terms. Characteristics were then integrated into a synthesis table, where overlap and differences could be more readily identified and described. Review and analysis of the terms and how their characteristics were described, was conducted in order to synthesise categories for resource withdrawal terms with definitions of prominent terms (step 10). Due to the nature of the review, namely analysing researcher and policy-maker use of resource withdrawal terms (as opposed to results of their analysis), the quality appraisal of studies was deemed unnecessary. Step 11 was the dissemination of the results.

## Results

**Fig. 1:**
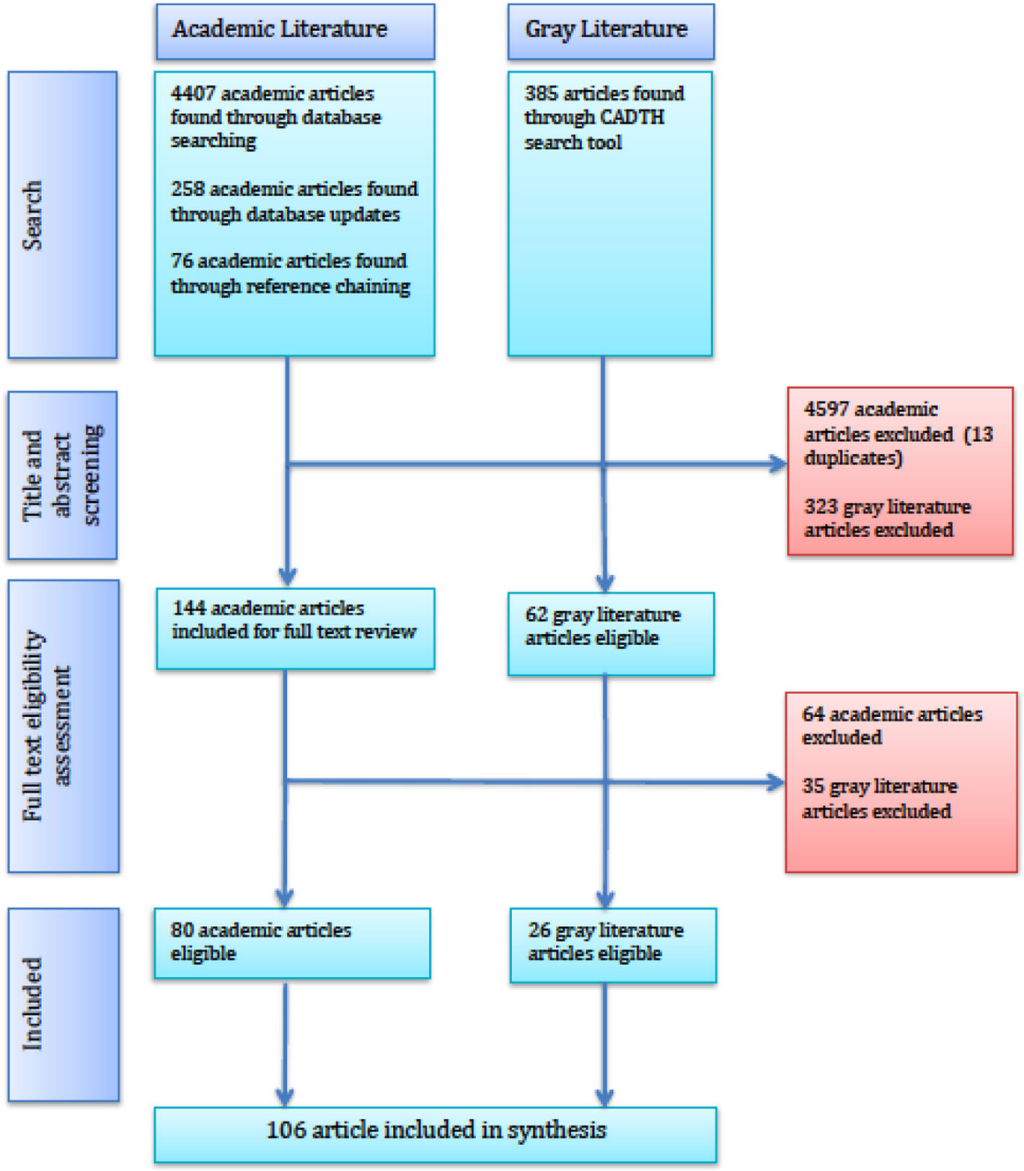
Systematic review process

Figure 1 illustrates the steps for the literature search and screening of both academic and grey literature databases. The academic literature search uncovered 4407 articles across all databases. Each database was reviewed sequentially, which meant duplicates were not removed until the full-text review stage. Title and abstract screening resulted in 146 eligible articles. At this stage, all eligible articles were combined and 13 duplicates were found and removed, leaving 133 articles for full-text review. Of these, 33 articles met eligibility criteria. Reference checking was used to identify another 36 eligible articles. Periodic updates of newly released articles that met search criteria from the electronic databases resulted in 11 additional eligible articles. As a result, 80 academic articles were included. The Canadian Agency for Drugs and Technology in Health tool for searching grey literature resulted in 385 articles, of which 26 were eligible for inclusion. In total, 106 articles met the eligibility criteria and were included in the analysis.

Additional file 1: Appendix 1 details how authors have used the same characteristics to refer to different resource withdrawal terms. From these results, it is evident that there has been extensive overlap of, confusion surrounding and contradiction between two prominent terms – (1) rationing and (2) disinvestment. The remaining terms identified in Additional file 1: Appendix 1 and Additional file 2: Appendix 3 have been categorised as policy options and are discussed below.

### Resource withdrawal characteristics

**Fig. 2:**
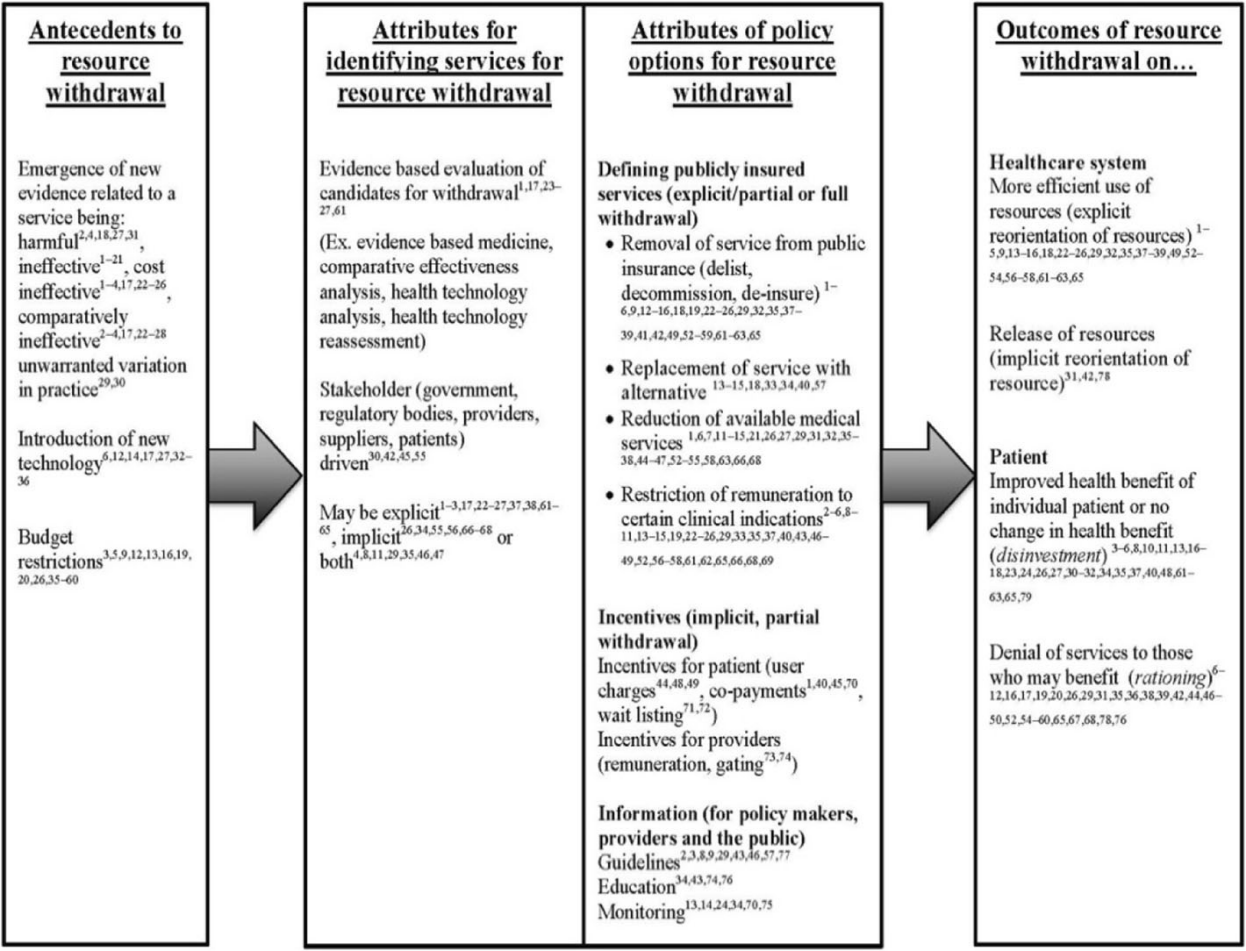
Characteristics of government-led resource withdrawal from medical services

Findings from the present study help categorise various resource withdrawal characteristics into antecedents, attributes and outcomes. Figure 2 depicts all the prominent descriptions of resource withdrawal found in the literature and organises them into their associated characteristics. Within each column, the prominent description of each resource withdrawal characteristic is identified. The process began with antecedents that have been identified to logically precede resource withdrawal, which was followed by the various processes that governments have used to identify medical services and the policy options to withdraw resources. Finally, there were the outcomes of withdrawal, which we have categorised as either patient or health system outcomes. The main components of each characteristic that were identified in the table are described in each section below.

#### Antecedents to resource withdrawal

Antecedents, often referred to as ‘triggers for resource withdrawal’, are events that proceed and logically lead to resource withdrawal. Additional file 1: Appendix 1 includes all antecedents found in the search. It is important to note that the articles described many of the antecedents as potential precursors but lacked analysis aimed at uncovering the underlying reasons for resource withdrawal. Antecedents were characterised into one of three categories – (1) emergence of new research evidence; (2) introduction of a new technology; or (3) budget restrictions.

##### Emergence of new research evidence

Articles described research evidence as a theoretical trigger for resource withdrawal but there was not a clear example of resource withdrawal being initiated by the emergence of new evidence. The following evidence-based antecedents have the potential to precede resource withdrawal: discovery that a service is unsafe or harmful; discovery that a service is ineffective; discovery that a service is effective but has poor cost-effectiveness; discovery that a service is comparatively ineffective; or discovery that there is evidence of regional variation in the use of a medical service [18, 26, 29, 38–41]. The observed lack of examples demonstrating resource withdrawal that resulted from the emergence of evidence may be attributed the ‘black box’ that encompasses much of public policy decision-making surrounding government-led resource withdrawal [42, 43]. This possibility speaks to the difficulty of evidence-based decision-making in resource withdrawal and the ambiguity found in evidence for clinical effectiveness [44]. Although this search failed to identify an explicit example of emerging evidence immediately preceding a process that led to government-led resource withdrawal, emerging research evidence was assumed by many to be the standard gateway to an explicit identification, assessment and implementation of resource withdrawal.

##### Introduction of a new technology

New technologies that were adopted into benefits packages for publicly insured services, whether more cost-effective or not, were viewed as an important prompt to replace existing technologies. Recent evidence from Prasad [45, 46] indicated that a large proportion of new technologies replace existing ones, despite the lack of clinical evidence that the novel technologies are superior. Prasad identified over 140 medical services that have been superseded by new technologies despite evidence that the previous service was more clinically effective. Adopting a new technology with a lack of supportive evidence illustrates the pressure that health systems are often under to adopt the ‘latest and greatest’ technologies and services. Perhaps most importantly, the introduction of new technology may lead to an implicit disinvestment from existing services, also referred to as obsolescence, rather than explicit policy-led resource withdrawal.

##### Budget restrictions

Budgetary events were the most cited trigger for resource withdrawal. This category included financial considerations that influence withdrawal, including a reduction in the overall healthcare budget, overspending and spending audits. Many researchers viewed cost savings as a likely goal of resource withdrawal [3, 17, 42] and some measured potential cost savings [25]. None, however, reported measured cost savings within the health system. Many of the services targeted for resource withdrawal for budgetary reasons were services for which medical necessity was contested, for example, cosmetic breast surgery [47] and in vitro fertilisation [19, 48, 49]. Importantly, cost was rarely the sole reason for service removal. Instead, cost-effectiveness was often used as a measure to help support withdrawal decisions.

#### Attributes for identifying services for resource withdrawal

Attributes of resource withdrawal relate to the process of implicitly or explicitly identifying and assessing services for withdrawal. The process has several phases, none of which are clearly detailed in the execution of government-led resource withdrawal. Despite the lack of detailed examples, recommendations for optimising evidence-based approaches are made throughout the literature. Approaches that have been recommended for identifying services well-suited for resource withdrawal include assessments of clinical effectiveness from EBM, CER, value assessment (HTA/HTR) and stakeholder consultation [5, 23, 25, 47, 50]. Henshall [51] reports that experts from the Health Technology Assessment International policy forum recommended that services be prioritised using robust evidence regarding their impact on patient health. Other scholars recommend engaging in resource withdrawal from services that have ‘low-value’ in the healthcare system. The latter measure may be problematic since the threshold for what constitutes as ‘low-value’ may differ between health systems [21]. Involving stakeholders throughout the withdrawal process, especially members of the public and healthcare service providers, was viewed as an essential factor in determining the success of a given resource withdrawal [3, 6, 17, 52, 53].

Processes are either explicit or implicit. Although research described governments recommending explicit resource withdrawal processes, most government resource withdrawal was actually implicit. Explicit processes of resource withdrawal include decisions made concerning the amount and forms of resources that are available, the recipients of resources, and the conditions under which resources will be received [54–56]. In the reviewed literature, explicit resource withdrawal was expected to involve significant political resistance because of its traceability [54, 57]. Implicit decision-making, on the other hand, typically occurs at the micro or meso level, without involving direct government decisions regarding resource withdrawal from a specific service [54]. Instead, governments will often enact policies that encourage implicit, indirect withdrawal at an organisational or provider level, and these processes are frequently driven by changes in medical practice or reductions in the budget over time [50].

#### Attributes of policy options for resource withdrawal

Many of the resource withdrawal terms used in the literature were associated with policy options for withdrawal. In some cases, terms such as decommissioning, delisting and de-insuring were used interchangeably with each other [26, 29, 58]. The policy option chosen will directly affect the overall impact of resource withdrawal on the system and patients, but the policy options do not represent the final consequence of the resource withdrawal. To clarify the use of the terms, we classify policy options into the following categories: (1) defining insured services, (2) incentives and (3) information.

Firstly, policy options may involve the complete or partial withdrawal of resources, which can be considered absolute and relative withdrawal, respectively [16, 17, 22, 47, 59]. Full withdrawal refers to the complete removal of governmental resources from a service, thereby eliminating the service’s availability through public provision or insurance. Partial withdrawal involves a reduction in resources provided to a service, thus reducing the accessibility of the service through public provision or insurance. Whether a given instance of resource withdrawal is categorised as full or partial depends largely on the policy option selected for implementation.

##### Defining insured services (explicit/partial or full withdrawal)

In the literature analysed, the most frequently referenced policy option was ‘defining health services’. Defining insured services is a direct, explicit method of resource withdrawal because it alters the manner through which public funds are provided for a service. Many of the terms used in the literature search were associated with defining insured services. These included the terms delisting, decommissioning, de-insuring and restricting. Defining insured services may include removal of services from the public benefits; replacement of the service with an alternative; reduction of the amount of service allowed to be provided; restricting provision or remuneration for a service to a specific patient or disease characteristic(s); and restricting provision or remuneration of a service to a specific type of healthcare providers and healthcare settings.

##### Incentives

Two categories of policy option incentives were identified in relation to resource withdrawal – (1) incentivising patients and (2) incentivising providers. Both types of incentivising are implicit, partial withdraw approaches because the choice of providing or using services remains in the hands of the provider or patient. Patients may be incentivised to avoid accessing services through user charges, co-payments or wait listing. Many countries have already instituted increased user charges for pharmaceuticals, primary care, specialist care, emergency care, inpatient care and long-term care. Despite these changes, the extent to which these disincentives are applied to ‘low-value’ or inappropriate services is unclear [60]. Provider behaviour may be incentivised through altering remuneration levers, especially through the modification of billing amounts for specific services [41]. ‘Gating’ is another way to disincentivise providers by increasing the administrative difficulty associated with providing a given service. One example of gating was described as the removal of two lab test check boxes for a vitamin B12 and serum ferritin tests, from a laboratory order form such that providers had to manually request the test (instead of just checking the box) [61].

##### Information (for policy-makers, providers and the public)

The provision of information to certain stakeholders has the potential to dissuade them from using targeted medical services. Forms of information provision aimed at implicitly withdrawing resources include the following:

- The provision of guidelines for providers to follow while delivering care. Violating specific guidelines, whether mandatory or recommended, may result in serious repercussions beyond the denial of reimbursement. Guidelines are not generally mandatory but provide a critical informational source for best practice [41, 47, 55, 60].
- The education of the public and providers about inappropriate and ‘low-value’ care effectiveness in order to reduce service reliance is not addressed in the articles analysed, although others have reported its futility [62]. Providing information on best practices and formal training, on the other hand, has resulted in an increased reliance on higher quality care [60]; and
- The monitoring of medical service use, including HTR, in order to evaluate the existing services further and provide additional information on best practice.

### Resource withdrawal outcomes: impact on system and patient

#### Impact on the healthcare system

The impact that resource withdraw ultimately has on a given healthcare system will depend on where, if anywhere, the withdrawn resources are reallocated to. There is limited evidence that deliberate reallocation of withdrawn resources occurs [4, 29, 41, 58]. One process that aims to reinvest resources through a systematic process is programme budgeting and marginal analysis [17, 43, 63]; however, results are mixed [63]. The reallocation of withdrawn resources was primarily associated with the term disinvestment. Although resource withdrawal may actually benefit the system by reallocating resources to more efficient services, the more likely scenario is that the withdrawal ‘frees up’ resources for use elsewhere but fails to predetermine where those resources will go [16]. In this scenario, it is impossible to determine if the resources will be used for more cost-effective services or if they end up being reallocated at all. It will be important for future research to examine whether resource withdrawal from inappropriate services achieves some form of improvement in health system performance.

#### Impact on the patient

The impact of resource withdrawal on the patient may be the most critical outcome. Patient health can, in essence, be conceptualised as the ‘ultimate dependent variable’, since improving the health of patients is the overall goal of the healthcare system [64, 65]. The impact that resource withdrawal has on patients’ health depends largely on the clinical effectiveness of the specific service and on the presence, or lack thereof, of a suitable alternative service. There are three potential outcomes of providing a service to a patient – (1) no change in patient health, (2) improvement in patient health or (3) deterioration in patient health. Resource withdrawal from a service that is inappropriate or expected to do more harm than good will ultimately benefit a given patient’s expected health outcomes. Resource withdrawal from a service that is appropriate or expected to do more good than harm will withhold potentially beneficial care from the patient, thus worsening their health. Exceptions exist when a medically optimal alternative service is made available.

### Resource withdrawal terms

Terms that had enough description and application to synthesise are included in Additional file 1: Appendix 2, along with their associated definition, attributes, antecedents, outcomes and health service type (when identified). Information in this appendix demonstrates the plethora of definitions used for several resource withdrawal terms. Twenty-three different definitions were found for disinvestment, 21 for rationing, 4 for decommissioning, 4 for delisting, 3 for HTR, 1 for de-insuring and 1 for de-implementation.

As Table 1 demonstrates, the terms rationing and disinvestment have had overlapping definitions used to describe them, with certain characteristics included in some definitions and excluded from others. Some researchers, for example, have described disinvestment as a form of rationing. Elshaug et al. [9] states, “*for the clinician there is often concern that disinvestment represents a blunt instrument of rationing*”. Decision-makers involved in determining healthcare funding arrangements have also used the terms interchangeably [73].

**Table 1:** Example of the overlap between the definitions of disinvestment and rationing

Description of definition	Disinvestment	Rationing
A process of selecting and reducing/removing select medical services	The formal processes and mechanisms that are used to reduce or discontinue the use of selected procedures and treatments [4]	Explicit decisions about the amounts and types of resources to be made available, eligible populations, and specific rules for allocation [54]
A process of selecting and reducing/removing only harmful/inefficient or ineffective medical services	The cessation or restriction of potentially harmful, clinically ineffective or cost inefficient practices [23] Taking resources from services that provide little or no value [66]	The elimination or reduction in the provision of a service based on evidence of low value [67] Limiting the choice of services to provide in an area with scarce resources; choice is decided on effectiveness, equity and patient choice [68]
A process of withdrawing resources and reallocating them to medical services of higher value	The processes of (partially or completely) withdrawing health resources from any existing healthcare practices, procedures, technologies or pharmaceuticals that are deemed to deliver little or no health gain for their cost and are thus not efficient health resource allocations; within this is the view to reallocation or reinvestment towards technologies, practices and programmes with greater demonstrated (cost-)effectiveness [9, 21, 29, 41, 52, 69, 70];	A priority-setting activity where resources are removed from the service such that other more effective ones are prioritised [71]
Restriction of medical services to only those who benefit	Funding decision to restrict the use of a service to those who may benefit the most [47]	Restriction of services to those who have a higher perceived benefit [72]

Several of the terms identified have been organised into the characteristics of resource withdrawal. De-listing, decommissioning, de-insuring and de-implementation have been organised as attributes of government policy options that redefine public health insurance packages, as displayed in Fig. 2. HTR is organised as an attribute in the process for identifying existing medical services.

### Defining prevalent resource withdrawal terms

The final objective of the present study is to refine definitions of prevalent, commonly used resource withdrawal terms. As Additional file 1: Appendix 2 illustrates, rationing and disinvestment are two foundational terms that represent the outcomes of resource withdrawal as opposed to interventions for identifying services (e.g. HTR) and policy options (e.g. de-listing). Table 1 provides examples of the prominent definitions of the two terms and illustrates the extensive overlap between their uses. Our approach to refine the definitions of these foundational terms was to synthesise their most common characteristics to form the basis for their respective definitions.

As described in the majority of the literature, rationing has the underlying presumption of scarce resources; therefore, rationing involves the prioritisation of resources resulting in some services being excluded from public funding, thereby denying people potentially beneficial services. In other words, if there were unlimited resources, there would be no need to ration. In comparison, disinvestment has arisen as an approach to reducing ineffective, harmful or ‘low-value’ medical services to improve the health of patients. Definitions ought to focus on these defining characteristics of rationing and disinvestment and namely on the impact on patient health. In order to determine the impact on patient health, the clinical effectiveness definition of appropriate care has been employed. This definition defines a service as appropriate if the benefits of providing a service sufficiently exceed the risks associated with the treatment [12]. Given this, the following definitions are suggested:

- Disinvestment: the full or partial withdrawal of resources from a medical service that it is clinically expected, on average, to result in a patient achieving health benefits or no change in health benefit.
- Rationing: the full or partial withdrawal of resources from a medical service that is clinically expected, on average, to result in a patient achieving diminished health benefits.

There are various methods to measure either condition-specific or patient-specific health outcomes and it is beyond the objectives of this study to propose an ideal measure. Hundreds of specific tools have been developed to measure health outcomes such as disease progression, patient survival time, patient satisfaction and quality of life. Using these definitions, if resource withdrawal worsens any of these health outcomes, it is an example of rationing healthcare. If resource withdrawal does not worsen an outcome, then it can accurately be defined as disinvestment. Mapping whether or not resource withdrawal from a specific medical service affects all of these categories requires the application of a complex model that may not be fully established yet. Despite this, existing measurements of particular health outcomes for medical services will have to be sufficient until new ones become available.

## Discussion

This study addressed the confusion between the use of various terms describing government-led resource withdrawal and the characteristics associated with those terms found in the academic and grey literature. In addition to Fig. 2, which encapsulates prominent descriptive characteristics of resource withdrawal, we have provided clarifying definitions of two of the most prominent and confused resource withdrawal terms – disinvestment and rationing. A key finding of our results is that characteristics of resource withdrawal have been associated with various terms in such a manner that none of the terms hold a consistent meaning for any of the characteristics. Although this finding is concerning, it is not unusual for terms and their associated characteristics to change over time or between different contexts [74]. Changes may serve as a way to map shifts in research trends that occurred when disinvestment emerged as a resource withdrawal term in healthcare.

Much of the inconsistent use of resource withdrawal terms has resulted from the differences between the traditional understanding of rationing and the emergent characteristics of disinvestment. Until approximately 2006, nearly all resource withdrawal activities described in the literature were considered a form of rationing. Disinvestment became increasingly popular in 2004, after the National Institute of Clinical Evidence (NICE) identified it as a priority activity for reducing spending and improving healthcare efficiency through the provision of appropriate care [64]. Some scholars attribute this term’s rise in popularity to the prominence of HTA [4, 19, 29]. As illustrated by the results of the present study, the use of disinvestment has been far from consistent and it is evident that the term borrowed some of the characteristics previously assigned to rationing, including some researchers defining it as an evidence-based process and a cost-cutting procedure [20].

A second key finding is that much of the existing literature primarily defines resource withdrawal terms, specifically rationing and disinvestment, based on either its antecedents or its attributes (see Additional file 1: Appendices 1 and 2). Government-led resource withdrawal is a public policy decision [75–78]; therefore, we argue that using these two characteristics as part of the definition will lead to more confusion because public policies have many contextual factors, beyond research evidence, which influence political decision-making [79, 80]. Clinical and economic evidence is rarely enough to put issues onto a governmental agenda. Instead, a myriad of factors affect the likelihood that certain public policy decisions will be prioritised [81]. The stated reasons for any government decision may not reflect the true policy objectives but instead represent a strategic use of language. As a result, there may be many underlying ‘true’ but unstated motivations. For example, a specific instance of resource withdrawal may be the result of a disagreement between various stakeholders on costs and benefits [82, 83]. This may be exacerbated by the perception that withdrawing resources from medical services is a risky political decision [29, 41, 55].

Furthermore, the use of process characteristics within the definition of a resource withdrawal term may also lead to continued confusion, especially since the process occurs at a public policy level. As our findings suggest, processes for assessing and recommending services for resource withdrawal are rarely straightforward [20] and many resource withdrawal decisions are politically or socially motivated. Influences, which can range from stakeholder input to interest group advocacy, become increasingly important in public policy decisions compared to clinical decision-making [80]. Protocols and guidelines for decision-making fail to invariably predict outcomes and often omit important contextual influences on the outcome [84]. Countries also have different institutional structures that have a high level of influence on which decisions end up on the government’s agenda and how these decisions are eventually formulated [85, 86]. For these reasons, the same withdrawal decision may indeed be made through a different process. If different processes can be used for a single definition, then the term no longer has clear boundaries and will likely not be consistently applied.

### Strengths and limitations

The primary strength of this study is the breadth of grey and academic literature that was analysed, which provided a plethora of documents from a variety of disciplines, countries and sources. The literature analysed included qualitative and quantitative studies, essays, editorials and literature from HTA, priority-setting economics and other social science disciplines. Many of the reviews that were included in the present study focused on resource withdrawal from inappropriate or low-value services [4, 41, 87], which excluded some government-led resource withdrawal from services that may be beneficial but not a high enough priority to be included in a public benefits package [47, 88]. The present study included all types of government-led resource withdrawal, which provided additional literature to analyse.

This study is limited to government-led resource withdrawal associated with medical services specifically and excludes withdrawal related to system reforms, organisational withdrawal and clinical decisions regarding withdrawal. Many reforms that include the redistribution of resources were not included in this study, including home care reform and mental health reform. Therefore, caution should be exercised if results are interpreted in other resource withdrawal contexts (i.e. beyond government-funded medical services). There is also a language bias, as only English studies are included. Furthermore, only OECD countries were included in the analysis and therefore implications may not apply to low-income countries. We were not interested in the results of each study included in the review; instead, we were interested in their use of terms and characteristics and therefore the objectives of the study did not require the collection and reporting of AMSTAR [89] items, including listing all studies that were not eligible, assessing the quality of evidence and combining findings from studies. Furthermore, the results are based on a limited range of resource withdrawal decisions and should not necessarily be applied to resource withdrawal implementation without further study. Policy decisions should not be automatically considered as implemented, as several other factors are known to influence implementation, which this study did not explore.

### Implications for research

The major implication of our findings is the suggestion to researchers to define resource withdrawal using the impact that a given service has on patient health. This will help set a standard for resource withdrawal terms and focuses on ‘value’ for the patient. The definitions presented here do not require measurement of the expected health benefit gained or denied, which would mandate a choice of measurement to determine a threshold (e.g. quality-adjusted life years). Instead, these revised definitions require evidence that the withdrawn service was expected to provide some benefit or not (through EBM) or to provide the most benefit from a set of alternatives as determined by CER. Two general scenarios exist – (1) the service provides benefit but is withdrawn (rationing) or (2) the service provides no benefit, may do harm, or is inferior to an alternative service option and is withdrawn (disinvesting).

## Conclusion

Following the rise of EBM, and subsequently HTA, government-led explicit resource withdrawal from medical services has attracted substantial attention. Resource withdrawal from inappropriate services may have a significant impact on the improvement of value in resource spending within healthcare systems. It is likely, however, that resource withdrawal will not achieve its full potential until its characteristics and prominent terms are used accurately and reliably. We present a clarifying framework (Fig. 2) that identifies the main characteristics of resource withdrawal and organises them. Furthermore, the established definitions presented here provide a step in the direction of clarifying resource withdrawal terms that are used in the academic terminology used to study policy-making. Although primarily intended as a theoretical contribution, results have some potential to promote accountability by governments, organisations and individuals responsible for resource withdrawal decisions at each level by emphasising the ultimate goal of the health system, that is, providing health benefit to patients. It is known that health systems are historically resistant to system-level reform [90]. By focusing the discussion on potential health benefits, this analysis may help shift focus away from basing public policy decisions on cost and towards improving patient health.

## Supplementary information

**Additional file 1: Appendix 1.** Characteristics of resource withdrawal terms used in the literature. **Appendix 2.** Table of definitions used for resource withdrawal terms.

**Additional file 2: Appendix 3.** References for the Fig. 1: Characteristics of resource withdrawal.

## Data Availability

All available data is included in the supplementary information files.

## Abbreviations

CER: Cost effectiveness research
EBM: Evidence-based medicine
HTA: Health technology assessment
HTR: Health technology reassessments
OECD: Organisation for Economic Co-operation and Development
