# Supplementary material for: Conceptualising characteristics of resources withdrawal from medical services: a systematic qualitative synthesis
Source: Health Res Policy Syst. 2020 Oct 28;18:123. doi: 10.1186/s12961-020-00630-9 (PMC7592573; doi:10.1186/s12961-020-00630-9)
Supplement: Supplementary file 1 — Additional file 1: Appendix 1. Characteristics of resource withdrawal terms used in the literature. Appendix 2. Table of definitions used for resource withdrawal terms. [file 12961_2020_630_MOESM1_ESM.docx]

**Supplement: Chapter 1**

Appendix 1: Characteristics of resource withdrawal terms used in the literature

|  | Efficacy on Service | | |  | Effect on System | | | | | Effect on patient | |
| --- | --- | --- | --- | --- | --- | --- | --- | --- | --- | --- | --- |
| **TERM: DISINVESTMENT** | Restricted use | Reduced use | Removed | Replaced | Reduced spending | Improved efficiency | Reallocation of resources (no clear effect) | Reduced benefit package | Improved quality of care | Denied access to beneficial services | Improved access to beneficial services |
| (Gallego, Haas, Hall, & Viney, 2010) |  | ✓ | ✓ |  |  |  |  |  |  |  |  |
| (Ibargoyen-Roteta, Gutiérrez-Ibarluzea, & Asua, 2010) | ✓ |  | ✓ |  |  | ✓ |  |  |  |  |  |
| (Pearson & Littlejohns, 2007) | ✓ |  | ✓ |  |  | ✓ |  |  |  |  | ✓ |
| (Rumbold, Allen, & Harris, 2008) |  | ✓ |  |  |  | ✓ |  |  |  |  | ✓ |
| (National Health Commitee & Commitee, 2012) | ✓ |  | ✓ |  |  | ✓ |  |  |  |  | ✓ |
| (Elshaug, Watt, et al., 2009)(Elshaug et al., 2008, 2007; Elshaug, Watt, Mundy, & Willis, 2012; Elshaug, Moss, et al., 2009; Garner & Littlejohns, 2011; Gerdvilaite & Nachtnebel, 2011) | ✓ |  | ✓ |  |  | ✓ |  |  |  |  | ✓ |
| (Daniels et al., 2013) | ✓ |  | ✓ |  |  | ✓ |  |  |  |  | ✓ |
| (Leggett et al., 2012) |  |  | ✓ |  |  | ✓ |  |  |  |  | ✓ |
| (Polisena et al., 2013) | ✓ |  | ✓ |  |  | ✓ |  |  |  |  | ✓ |
| (Schmidt, 2010) | ✓ | ✓ | ✓ |  |  | ✓ |  |  |  |  | ✓ |
| (Haas, Hall, Viney, & Gallego, 2012) | ✓ | ✓ | ✓ |  |  | ✓ |  |  |  | ✓ |  |
| (García-Armesto, Campillo-Artero, & Bernal-Delgado, 2013) |  | ✓ | ✓ |  | ✓ | ✓ |  |  |  |  | ✓ |
| (Watt et al., 2011) |  | ✓ | ✓ |  |  | ✓ |  |  | ✓ |  | ✓ |
| (Nuti, Vainieri, & Bonini, 2010) |  | ✓ | ✓ |  | ✓ | ✓ |  |  |  | ✓ |  |
| (Haines et al., 2014) |  |  | ✓ |  |  | ✓ |  |  |  | ✓ |  |
| (Karnon et al., 2009) | ✓ |  |  | ✓ |  | ✓ |  |  |  |  |  |
| (Frønsdal et al., 2010) |  |  |  | ✓ |  | ✓ |  |  | ✓ |  | ✓ |
| (Hodgetts et al., 2014) | ✓ |  |  | ✓ |  | ✓ |  |  |  |  | ✓ |
| (Joshi, Stahnisch, & Noseworthy, 2009) |  | ✓ |  |  | ✓ | ✓ |  |  |  |  | ✓ |
| (Donaldson, Bate, Mitton, Dionne, & Ruta, 2010) |  | ✓ |  |  |  |  | ✓ |  | ✓ | ✓ | ✓ |
| (Robinson et al., 2013) |  |  | ✓ |  |  |  | ✓ |  |  | ✓ |  |
| (Mitton, et al. 2011) |  |  | ✓ |  |  |  | ✓ |  |  | ✓ |  |
| (Russell et al., 2014) | ✓ |  |  |  | ✓ | ✓ |  |  |  |  | ✓ |
| **TERM: RATIONING** |  |  |  |  |  |  |  |  |  |  |  |
| (Moreira, 2011) |  | ✓ |  |  | ✓ |  |  |  |  | ✓ |  |
| (Martin, 2015) | ✓ | ✓ | ✓ |  |  | ✓ |  |  |  | ✓ | ✓ |
| (Bevan & Brown, 2014) |  | ✓ |  |  |  | ✓ |  |  |  | ✓ |  |
| (Aaron & Schwartz, 1990) |  | ✓ |  |  |  | ✓ |  |  |  | ✓ |  |
| (R Klein et al., 1995; R Klein, 1995; Rudolf Klein, 1994) | ✓ | ✓ |  |  |  | ✓ |  |  |  | ✓ |  |
| (Meadowcroft, 2008) |  | ✓ |  |  | ✓ |  |  |  |  | ✓ |  |
| (D. Mechanic, 1997; David Mechanic, 1995) | ✓ | ✓ |  |  |  |  | ✓ |  |  | ✓ |  |
| (Gerdvilaite & Nachtnebel, 2011) |  | ✓ |  |  | ✓ |  |  |  |  | ✓ |  |
| (Gravelle & Siciliani, 2007) | ✓ | ✓ |  |  |  | ✓ |  |  |  | ✓ | ✓ |
| (Carlsson, 2010) | ✓ |  |  |  |  | ✓ |  |  |  | ✓ | ✓ |
| (Giacomini, 1999) |  | ✓ |  |  |  | ✓ |  |  |  |  |  |
| (Syrett, 2003) | ✓ |  | ✓ |  |  | ✓ |  |  |  | ✓ |  |
| (Maxwell, 1995; Schwartz & Mendelson, 1992) | ✓ |  | ✓ |  |  | ✓ |  |  |  | ✓ |  |
| (Fox & Leichter, 1991) | ✓ |  |  |  | ✓ | ✓ |  |  |  | ✓ | ✓ |
| (Rosenthal & Newhouse, 2002) | ✓ | ✓ |  |  |  | ✓ |  |  |  | ✓ | ✓ |
| (Griffiths, 2002) | ✓ |  | ✓ |  |  | ✓ |  |  |  | ✓ | ✓ |
| (Mullen, 1998) | ✓ | ✓ | ✓ |  |  | ✓ |  |  |  | ✓ |  |
| (Redmayne & Klein, 1993a) |  | ✓ | ✓ |  |  | ✓ |  |  |  | ✓ |  |
| (Campbell, 1995) |  | ✓ | ✓ |  |  | ✓ |  |  |  | ✓ |  |
| Malone, 1998 |  | ✓ | ✓ |  | ✓ |  |  |  |  | ✓ |  |
| (Hope, Hicks, Reynolds, Crisp, & Griffiths, 1998) | ✓ |  | ✓ |  |  | ✓ |  |  |  | ✓ |  |
| (Plomer et al., 1999) | ✓ |  | ✓ | ✓ |  | ✓ |  |  |  | ✓ |  |
| (Rudolf Klein & Maybin, 2012) | ✓ | ✓ | ✓ |  |  | ✓ |  |  |  | ✓ |  |
| **TERM: DEINSURING** |  |  |  |  |  |  |  |  |  |  |  |
| (Giacomini et al., 2000; Giacomini, 1999) |  |  | ✓ |  |  |  |  | ✓ |  | ✓ |  |
| **TERM: Health Technology Reassessment** |  |  |  |  |  |  |  |  |  |  |  |
| (Leggett et al., 2012) | ✓ | ✓ | ✓ | ✓ |  | ✓ |  |  |  |  | ✓ |
| (Mackean et al., 2013) | ✓ | ✓ | ✓ | ✓ |  | ✓ |  |  |  |  |  |
| (Banta & Thacker, 1990) | ✓ | ✓ | ✓ | ✓ |  | ✓ |  |  |  |  |  |
| **TERM: Decommissioning** |  |  |  |  |  |  |  |  |  |  |  |
| (Robert, Harlock, & Williams, 2014) |  |  | ✓ | ✓ |  | ✓ |  |  |  | ✓ | ✓ |
| (Joshi et al., 2009) |  | ✓ | ✓ |  | ✓ |  |  |  |  |  | ✓ |
| (Robinson et al., 2013) |  |  | ✓ |  |  |  | ✓ |  |  |  |  |
| (Elshaug, Watt, et al., 2009) |  |  | ✓ |  |  |  |  | ✓ |  | ✓ |  |
| **De-implementation** |  |  |  |  |  |  |  |  |  |  |  |
| (Prasad & Ioannidis, 2014) |  |  | ✓ | ✓ |  | ✓ |  |  |  |  | ✓ |
| **De-List** |  |  |  |  |  |  |  |  |  |  |  |
| (Joshi et al., 2009) | ✓ |  |  |  |  | ✓ |  |  |  | ✓ |  |
| (Gordon et al., 2007; Kiran et al., 2013; Landry et al., 2006) |  |  | ✓ |  | ✓ |  |  |  |  | ✓ |  |
| (Elshaug, Watt, et al., 2009) |  |  | ✓ |  |  | ✓ |  |  |  | ✓ |  |
| (Landry et al., 2006) | ✓ | ✓ |  |  |  | ✓ |  |  |  | ✓ |  |

Appendix 2: Table of definitions used for resource withdrawal terms

| Definition of resource withdrawal concept | **Attributes**  Quality or feature characterizing concept | | | **Antecedents**  Events that precede resource withdrawal | | | | **Consequences/ Outcomes** | | | | | | | | | | | Service example (country) | | | Reference | | | | |  |  |
| --- | --- | --- | --- | --- | --- | --- | --- | --- | --- | --- | --- | --- | --- | --- | --- | --- | --- | --- | --- | --- | --- | --- | --- | --- | --- | --- | --- | --- |
|  |  |  |  |  |  |  |  | Effect on service | | | Effect on system | | | | Effect on patient | | | |  | | |  | | | | |  |  |
| TERM: DISINVESTMENT | | | | | | | | | | | | | | | | | | | | | | | | | |  |  |  |
| The formal processes and mechanisms that are used to reduce or discontinue the use of selected procedures and treatments. | Explicit process  System level funding decision  Evidence based | | | Evidence of ineffectiveness or outside the acceptable level of cost-effectiveness | | | | Reduced  Removed | | | Unspecified impacts (dependent on type of service chosen for disinvestment) | | | | Unspecified impacts (dependent on typo service chosen for disinvestment) | | | | Unspecified medical services | | | (Gallego et al., 2010) | | | | |  |  |
| The cessation or restriction of potentially harmful, clinically ineffective or cost inefficient practices | Explicit process  System level funding decision | | | Evidence of:  Harmfulness, ineffectiveness,  cost inefficiency, lack of safety,  lack of quality of health care,  poor risk/benefit ratio,  lack of evidence of efficacy | | | | Restricted  Removed | | | More efficient use of resources | | | | Improved health benefits | | | | Unspecified medical services  (Spain) | | | (Ibargoyen-Roteta et al., 2010) | | | | |  |  |
| An explicit process of taking resources from one service in order to use them for other purposes that are believed to be of better value. | Two stage process  Priority setting    Resource allocation  Explicit process  System level decision | | | Evidence of comparative ineffectiveness  Evidence of ineffectiveness or cost-ineffective  Budget reduction | | | | Replacement  Restricted (guidelines) | | | More efficient use of resources | | | | More beneficial care | | | | Unspecified medical services  (England) | | | (Pearson & Littlejohns, 2007) | | | | |  |  |
| A policy driven process of defunding services that provide less favourable health outcomes. | Policy driven  Evaluative process  Explicit  process  System level funding decision  Full or partial withdrawal | | | Evidence of:  Unsafe, harmful, ineffective, low health gain, or cost ineffectiveness  Evidence of comparative ineffectiveness | | | | Defunding leading to a reduction of service availability | | | Release of resources for more efficient use | | | | More beneficial care | | | | Unspecified medical services,  (New Zealand) | | | (Rumbold, Allen, & Harris, 2008b) | | | | |  |  |
| Withdraw or reduce an investment of services in order to better allocate resources to services that provide more benefit. | Implicit or explicit process  Priority setting | | | Evidence of:  Safety, clinical effectiveness, cost effectiveness | | | | Full or partial removal | | | More efficient use of resources | | | | More beneficial care | | | | Unspecified medical services  (New Zealand) | | | (National Health Committee, 2012) | | | | |  |  |
| The processes of (partially or completely) withdrawing health resources from any existing health care practices, procedures, technologies, or pharmaceuticals that are deemed to deliver little or no health gain for their cost and thus are not efficient health resource allocations. Within this is the view to reallocation or reinvestment towards technologies, practices, and programs with greater demonstrated (cost) effectiveness. | Two stage process (withdrawal, reinvest)  Prioritization  Evidence based process  Explicit  process  System level funding decision  Full or partial withdrawal | | | Evidence that a service is comparatively ineffective or provides no benefit | | | | Full or partial removal of service from insurance scheme | | | More efficient use of resources | | | | More beneficial care | | | | Unspecified medical services  (Australia, Spain, Italy, Canada) | | | (Elshaug, Watt, et al., 2009)(Elshaug, Moss, et al., 2009; Elshaug et al., 2008, 2007, 2012; Garner & Littlejohns, 2011; Gerdvilaite & Nachtnebel, 2011) | | | | |  |  |
| Full withdrawal of services and interventions in order to use them for other purposes that are believed to be of better value | Two-stage process (withdrawal, reinvest) | | | Budgetary shortfalls  Service reductions due to inappropriateness  Benchmarking (e.g. lower cost for the same output).” | | | | Full withdrawal (full decommissioning of a service)  Restriction of service (guidelines or fee schedule changes for patient subgroups)  Retraction  (investing in less of an intervention) | | | Service redesign  More efficient use of resources | | | | Receiving  ‘right care at the right time in the right way’ | | | | Unspecified medical services  (England) | | | (Daniels et al., 2013) | | | | |  |  |
| The removal of funding from services based on clinical ineffectiveness or financial inefficiency | Priority setting | | | Evidence of: ineffective services or financial inefficiency | | | | Removal of funding | | | More efficient use of resources | | | | More health benefit | | | | Unspecified medical services | | | (Leggett et al., 2012) | | | | |  |  |
| The complete or partial withdrawal of resources from healthcare services and technologies that are regarded as unsafe, ineffective or inefﬁcient, with those resources shifted to health services and technologies with greater clinical- or cost-effectiveness | Two stage process (withdrawal, reinvest)  Explicit  process  System level funding decision  Full or partial withdrawal | | | Stakeholder engagement (to determine best reallocation) | | | | Full or partial withdrawal | | | More efficient use of resources | | | | More health benefit | | | | Unspecified medical services | | | (Polisena et al., 2013) | | | | |  |  |
| Category 1: Reallocation of resources from services that are providing positive benefit to areas where greater benefit may be gained.  Category 2: Removal of interventions or services that are ineffective or provide little or no value for money spent.  Category 3: Combines categories 1 and 2 | Two part process (identification and withdrawal)  Explicit  process  System level funding decision  Full or partial withdrawal | | | Budgetary shortfalls  Evaluation of a service that is determined to be comparatively inferior (low value) | | | | Three potential options for disinvestment: 1) stop a treatment, intervention or service; 2) scale back a treatment, intervention, or service; or 3) replace a treatment, intervention or service with an alternative. | | | More efficient use of resources | | | | More health benefits | | | | Unspecified medical services | | | (D. Schmidt, 2010) | | | | |  |  |
| Processes by which a health system or service removes technologies, without necessarily replacing them | Implicit or explicit  System level funding decision  Full or partial withdrawal | | | Unwarranted variations in practice | | | | Dropping of services from basket  Discourage use through guidelines  Limiting availability  Restricting indications to patient subpopulation | | | More efficient use of resources | | | | Reduction in access to potentially beneficial services | | | | Unspecified medical services | | | (Haas et al., 2012) | | | | |  |  |
| Withdraw of funding from no or low value health interventions, with the intent to free up resources for reinvestment in superior services. | System level decision | | | Adoption of new technology | | | | Reduced/removed funding for service | | | More efficient use of resources  Reduced spending | | | | More beneficial care | | | | Various low value services  (Spain) | | | (García-Armesto et al., 2013) | | | | |  |  |
| Disinvestment seeks to improve quality of care and health outcomes by evaluating existing health services; identifying those that do not provide safe, effective or cost-effective care; and redirecting funding away from these services toward those with superior safety, effectiveness and/or cost-effectiveness proﬁles | Three stage process: identification, withdraw, reinvest  Evaluative process    System level process  Explicit | | | Cost effectiveness research | | | | Reduction or removal of services | | | Improved quality  More efficient resource allocation | | | | More beneficial care | | | | ART  (Australia) | | | (Watt et al., 2011) | | | | |  |  |
| Withdrawing health resources from existing healthcare practices, procedures technologies or pharmaceuticals that are deemed to deliver little or no health benefit | System level process  Explicit | | | Overspending  Cost Control issues | | | | Reduction or removal of service | | | More efficient use of resources  Lowered overall spending | | | | Potential denial of health benefit | | | | Various medial services  (Italy) | | | (Nuti et al., 2010) | | | | |  |  |
| Removal of resources from non-cost-effective practices, procedures, technologies, or medicines. | Evidence based identification and assessment  Reinvestment | | | Evidence of cost-effectiveness  Budget cuts | | | | Removal of service | | | More efficient distribution of resources | | | | Potential denial of health benefit | | | | Unspecified medical services  (Australia) | | | (Haines et al., 2014) | | | | |  |  |
| The freeing up of resources in order to fund another service. | Resource allocation | | | New technology adoption | | | | Expansion of new services, reduction in older service. | | | More efficient use of resources | | | |  | | | | Screening for amblyopia and strabismus (as examples)  (UK) | | | (Karnon et al., 2009) | | | | |  |  |
| Removal of resources at the end of a technological lifecycle, which begins with innovation and moves through adoption before reaching a stage where that innovation is no longer clinically or cost effective. | Passive process of obsolescence | | | Adoption of new technology | | | | Replacement of obsolete services | | | More efficient use of resources | | | | Stop access to unbeneficial care | | | | Unspecified medical services | | | (Frønsdal et al., 2010) | | | | |  |  |
| Redirecting funds from evaluated existing services that do not provide sufficiently safe, effective or cost-effective care to services deemed superior. | System level process  Evidence based process | | | Cost control measures  Quality control initiative | | | | Reduced use  Replacement | | | More efficient use of services | | | | Better health outcomes | | | | ART  (Australia) | | | (Hodgetts et al., 2014) | | | | |  |  |
| The displacement of non–cost-effective technologies for resource reinvestment or reallocation. | System level  Priority setting activity | | | New evidence of clinical or cost effectiveness  New technologies | | | | Reduced use | | | More efficient use of resources  Reduction in costs | | | | Better health outcomes | | | | Unspecified medical services | | | (Joshi, Stahnisch, & Noseworthy, 2009) | | | | |  |  |
| Taking resources from services the provide little or no value | Evidence based  Priority setting | | | Spending audit  Evidence of harm | | | | Reduction in use | | | Improved quality | | | | Better health outcomes  Denial of potentially beneficial care | | | | Unspecified medical services  (UK) | | | (C. Donaldson et al., 2010) | | | | |  |  |
| The removal of services that were once covered by public insurance | Priority setting | | | Budget cutting | | | | Service no longer available | | | Improved resource allocation | | | | Denial of potentially beneficial care | | | | Unspecified services  (UK) | | | (Robinson et al., 2013) | | | | |  |  |
| Removing resources from a service identified as lower value than others. | Stakeholder driven  Explicit process | | | Budgetary shortcomings | | | | Removal of service | | | Release of resources | | | | Denial of potentially beneficial care | | | | Unspecified medial cervices  (Canada) | | | (Mitton, et al. 2011) | | | | |  |  |
| Funding decision to restrict the use of a service to those who may benefit the most. | Evidence based on social judgment  Process of assessment  System level funding decision | | | Priority setting activities  Unwarranted variation  Budget shortfalls | | | | Removal of funds that restrict service availability | | | Reduced spending,  More efficient use of resources | | | | Improved access to those most in need, less access others | | | | Cosmetic surgical procedure (breast reduction)  (England) | | | (Russell et al., 2014) | | | | |  |  |
| **TERM: RATIONING** | | | | | | | | | | | | | | | | | | | | | | | | | | | |  |
| The use of institutional procedures for the systematic allocation of resources within health care systems. | Priority Setting  Resource Allocation  System level decision | | Evidence of effectiveness  Evidence of cost-ineffectiveness | | | | Limited availability of service | | | | | | Improved spending | Denial of potentially beneficial care | | | | Dementia drugs  (UK) | | | (Moreira, 2011) | | | | |  |  |  |
| The process of deciding which healthcare services are funded by government and which ones are not. | Explicit and implicit process  Evidence based appraisal  Priority setting  Explicit decision | | Budget reductions | | | | Defunding  Denying access  Selection/restriction  Deflection  Deterrence  Delay  Diluting | | | | | | More efficient resource allocation | Denial of access to potentially beneficial care  Improved quality of care | | | | Various medical services  (Australia) | | | (Martin, 2015) | | | | |  |  |  |
| The denial of health care that is beneficial but is deemed to be too costly. | Cost based process | | Budget shortage | | | | Reduced access | | | | | | More efficient spending | Denial of access to potentially beneficial care | | | | Various services in acute, chronic and end of life care | | | (Bevan & Brown, 2014) | | | | |  |  |  |
| The denial of commodities to those who have the money to buy them in order to eliminate inefficiencies. | Cost based process | | Introduction of new technology  Budget shaortage | | | | Reduced access | | | | | | More efficient use of resources | Denial of access to potentially beneficial care | | | | Unspecified services  (USA) | | | (Aaron & Schwartz, 1990) | | | | |  |  |  |
| Involves the denial or dilution of something that is potentially beneficial to the patient: he or she is getting less in the way of treatment than might be thought desirable in a world with unlimited resources. | Concerned with evidence of medical necessity  Implicit micro clinical decision | | Priority setting activities provide the parameters | | | | Restricted  Reduced | | | | | | More efficient use of resources | Denial of potentially beneficial services | | | | Unspecified medical services  (UK) | | | (R Klein, 1994, 1995; R Klein, Day, & Redmayne, 1995b) | | | | |  |  |  |
| Rationing involves depriving patients of care from which they may benefit and which they want | Value laden process | | Political event  Budget reductions | | | | Reduced availability | | | | | |  | Denial of potentially beneficial care | | | | Unspecified medical services  (UK) | | | (Meadowcroft, 2008) | | | | |  |  |  |
| Explicit decisions about the amounts and types of resources to be made available, eligible populations, and specific rules for allocation.  Implicit discretionary decisions made by managers, professionals, and other health personnel functioning within a fixed budgetary allowance. | Complex set of actions at various levels of health system  Implicit and explicit process  Clinical (implicit) decision on who receives care  System decision on resource allocation | | Budget reductions | | | | Restricted use  Reduced use | | | | | | Reallocation of resources | Denial of potentially beneficial care | | | | Queuing, reduction in services, substitution, elimination of services  (UK) | | | (Mechanic, 1995, 1997) | | | | |  |  |  |
| Withholding beneficial interventions for cost reasons | Cost related  Implicit decision  Micro level decision | | Budgetary reductions | | | | Reduced use | | | | | | Reduced spending | Denial of potentially beneficial care | | | | Unspecified medical services | | | (Gerdvilaite & Nachtnebel, 2011) | | | | |  |  |  |
| Restriction of services to those who have a higher perceived benefit. | Effectiveness based  Partial withdrawal  Micro level  Implicit decision | | Budget reductions | | | | Restriction  Reduction | | | | | | More efficient use of resources | Denial of potentially beneficial care  Access to more beneficial care | | | | Unspecified service  (UK) | | | (Gravelle & Siciliani, 2008) | | | | |  |  |  |
| Rationing is a priority setting activity where resources are removed from the service such that other more effective ones are prioritized. | Priority setting  Full withdrawal  Effectiveness based  System level  Explicit process  Implicit clinical decisions | | New evidence of effectiveness, cost effectiveness | | | | Restricted use | | | | | | More efficient use of resources | Denial of potentially beneficial care  Access to more beneficial care | | | | Extending time between follow-up visits, check ups  Self care or nursing visits for minor problems  Better compliance with existing guidelines.  (Sweden) | | | (Carlsson, 2010) | | | | |  |  |  |
| The allocation of scarce resources among competing claims | Priority setting  Resource allocation | | Budget reductions  Adoption of new services  Political reasons | | | | Reduced use | | | | | | More efficient of resources | Denial of potentially beneficial care | | | | Various specific examples  (Canada) | | | (Giacomini, 1999) | | | | |  |  |  |
| The elimination or reduction in the provision of a service based on evidence of low value. | Evidence based process  Full or partial withdrawal | | Budget reductions  New evidence of effectiveness or cost effectiveness  Political factors | | | | Removal or reduction | | | | | | More efficient resource allocation | Denial of potentially beneficial care | | | | Unspecified medical services  (England) | | | (Syrett, 2003) | | | | |  |  |  |
| Denying some patients access to certain types of expensive but useful services. | Spending basis | | Budget reductions | | | | Removal or reduction | | | | | | More efficient resource allocation | Denial of potentially beneficial care | | | | Chemotherapy  (UK) | | | (Maxwell, 1995; Schwartz & Mendelson, 1992) | | | | |  |  |  |
| Prioritizing services, which that may provide the most benefit if under a given budget. | Clinical and cost effectiveness assessment  Priority setting | | Budget reductions  Social events  Political events | | | | Restricting the type of beneficiary, the coverage, or creating disinvests for providers. | | | | | | Reduced spending  More efficient resource allocation | Access to more beneficial care  Denial of potentially beneficial care | | | | Specific 700 type of medical services  (US) | | | (Fox & Leichter, 1991) | | | | |  |  |  |
| The elimination of services that provide low value. | Explicit  system level priority setting  Cost effectiveness based  Implicit clinical decision making | | Evidence of cost ineffectiveness | | | | Reduced use  Restricted use | | | | | | More efficient resource allocation | Denial of potentially beneficial care  Improved quality of care | | | | Unspecified medical services  (US) | | | (Rosenthal & Newhouse, 2002) | | | | |  |  |  |
| Limiting the choice of services to provide in an area with scarce resources. Choice is decided on effectiveness, equity and patient choice. | Explicit evidence based decision making process | | Budget reductions | | | | Restricted  Removed | | | | | | More efficient resource allocation | Better patient care  Denial of potentially beneficial care | | | | Unspecified medical services  (England) | | | (Griffiths, 2002) | | | | |  |  |  |
| Presents various definitions without claiming a correct one.  Claims “the denial of non-effective or non-beneficial treatment does not constitute rationing” | Cost related process  Resource allocation/  Priority setting decision | | Budget reductions  Evidence of cost-ineffectiveness | | | | Deterrence  Delay  Deflection  Dilution  Denial | | | | | | More efficient use of resources | Denial of potentially beneficial care | | | | Unspecified medical services  (UK) | | | (Mullen, 1998) | | | | |  |  |  |
| Limiting resources available to provide services. | Based on a criteria (evidence, need, equity, low value for money)  Resource allocation | | Stakeholder input  Budget reductions | | | | Reduction or removal | | | | | | Reduced spending | Denial of potentially beneficial care | | | | In vitro fertilization (IVF)  (UK) | | | (Redmayne & Klein, 1993a) | | | | |  |  |  |
| Decision on what healthcare funds will be spent on, given limited resources. | Rational decision making | | Budget reductions  Political events | | | | Reduction or removal | | | | | | More efficient distribution of resources | Denial of potentially beneficial care | | | | Unspecified medical services  (New Zealand) | | | (Campbell, 1995) | | | | |  |  |  |
| The process by which prioritizing care is achieved through exclusion, denial, delay, termination of service or early discharge. | Implicit process  made at the clinical level  Political problem | | Budget reductions | | | | Reduction or removal | | | | | | Reduced spending | Denial of potentially beneficial care | | | | Unspecified medical services  (UK) | | | Malone, 1998 | | | | |  |  |  |
| Decisions about what to fund or what not to fund. | Principle based decision making process  (patient choice, effectiveness, equity)  Implicit decision | | Budget reductions  Evidence of ineffective treatments | | | | Restriction, or removal | | | | | | More efficient resource allocation | Denial of potentially beneficial care | | | | Various medical services including riluzole, isotretinoin | | | (Hope, Hicks, Reynolds, Crisp, & Griffiths, 1998) | | | | |  |  |  |
| Making choices about what are the most effective/ efficient type of health services to provide under a limited budget. | Evidence based (effectiveness)  Priority setting | | Budget reductions  Political events  Social events | | | | Reduction or removal  Replacement | | | | | | More efficient resource allocation | Denial of potentially beneficial care | | | | IVF  (England and Wales) | | | (Plomer, Smith, & Martin-Clement, 1999) | | | | |  |  |  |
| Decisions about which patients should be treated and how resulting in a deprivation of possible benefits for patient. | Resource allocation  Priority setting | | Budget reductions  Evidence of cost ineffectiveness | | | | Restriction (denial, selection, deterrence, deflection or dilution) or removal. | | | | | | More efficient resource allocation | Denial of potentially beneficial care | | | | Various medical services  Including Tonsillectomy, Bariatric surgery, IVF  (UK, gray literature) | | | (Rudolf Klein & Maybin, 2012) | | | | |  |  |  |
| TERM: DEINSURING | | | | | | | | | | | | | | | | | | | | | | | | | |  |  |  |
| Removal of medical services from the physician fee schedule | | Full withdrawal  Policy driven process  System level | | | | Stakeholder input  Evidence of ineffectiveness  Budget reductions | | | | Removal from public insurance scheme (no longer publicly funded) | | | Less services available | | | | Denial of potentially beneficial care  Reduced choice | | | Various medical services  (Canada) | (Giacomini, 1999; Giacomini et al., 2000) | | | | |  |  |  |
| TERM: Health Technology Reassessment | | | | | | | | | | | | | | | | | | | | | | | | | |  |  |  |
| Health technology reassessment process includes identification, prioritization, evaluation, implementation and monitoring of health care services | | Evidence based  Identification, prioritization, evaluation, implementation and monitoring of technology | | | | Evidence of cost ineffectiveness  Evidence of ineffectiveness  Budgetary restrictions | | | | Withdrawal (full or partial of resources)  Replaced | | | More efficient use of resources | | | | Access to more care with an evidence base | | | Unspecified medical services  (international) | (Leggett et al., 2012) | | | | |  |  |  |
| A structured, evidence-based assessment of the clinical, social, ethical and economic effects of a technology currently used in the health care system, to inform optimal use of that technology in comparison to its alternatives. | | Evidence based process  Reinvestment | | | | Evidence of cost ineffectiveness  Evidence of ineffectiveness  Monitoring  Adoption of new technology | | | | Reduced  Restricted  Replaced  Removed | | | More efficient use of resources | | | | More education & information on services | | | Unspecified medical services  (international) | | | | | (Mackean et al., 2013) |  |  |  |
| Policy research that examines the short and long term clinical, societal, economic, ethical, and legal consequences of application of existing technologies | | Evidence based process  Reinvestment | | | | Evidence of cost ineffectiveness  Evidence of ineffectiveness  Monitoring | | | | Reduced  Restricted  Replaced  Removed | | | More efficient use of resources | | | | More education and & information on services | | | Various medical services including  Electronic fetal monitoring, hysterectomy,  Episiotomy, electroencephalography  (international) | | | | | (Banta & Thacker, 1990) |  |  |  |
| TERM: Decommissioning | | | | | | | | | | | | | | | | | | | | | | | | | | | |  |
| The practice of replacement and removal of health care services as an evidence based practice, including the policies to remove the interventions and reconfiguring of services. | | Policy driven  Evidence based | | | | Evidence of cost ineffectiveness  Evidence of ineffectiveness  Budget restrictions | | | | Replacement or removal | | | More efficient resource allocation | | | | Better health outcomes  Denial of potentially beneficial care | | | Various medical and nonmedical services including  drug therapies | | | | | (Robert, Harlock, & Williams, 2014) | | | |
| To remove from service | | Policy driven process  Assessment  Priority setting  Resource allocation | | | | Evidence of clinical or cost effectiveness  Adoption of new technology | | | | Removal, or reduction | | | Reduce spending | | | | Safer, more effective treatments | | | Unspecified medical services  (Canada) | | | | (Joshi et al., 2009) | | | | |
| Removing services from public payment | | Policy driven process | | | | Budget restrictions  Evidence of efficacy | | | | Closure of services | | | Improved spending  Service redesign | | | |  | | | Unspecified medical services  (UK) | | | | (Robinson et al., 2013) | | | | |
| Removal of services from an insurance schedule | | Priority setting  Resource allocation  Policy driven process | | | | Evidence of effectiveness  Introduction of new technologies | | | | Removal of service | | | Reduced benefit package | | | | Denial of potentially beneficial care | | | Unspecified medical services  (Canada) | | | | (Elshaug, Watt, et al., 2009) | | | | |
| De-implementation | | | | | | | | | | | | | | | | | | | | | | | | | | | |  |
| The abandonment of medical practices | | Evidence based decisions (including decisions based on lack of evidence) | | | Evidence of ineffectiveness,  Evidence of comparative ineffectiveness  or harm | | | | Removal of service  Medical reversal (replacement with superior service) | | | More efficient allocation of resources | | | | Improved health benefits | | | Various medical services  (US) | | | | (Prasad & Ioannidis, 2014) | | | | |  |
| De-List | | | | | | | | | | | | | | | | | | | | | | | | | | | |  |
| The discontinuation of a public insurance for a healthcare service | | Policy decision  Priority setting  Resource allocation | | | Evidence of ineffectiveness  Evidence of comparative ineffectiveness  Adoption of new technology | | | | Removal  Provision of better services | | | More efficient allocation of resources | | | | Better health outcomes | | | Unspecified medical services  (Canada) | | | | (Joshi et al., 2009) | | | | |  |
| Services that are partially or fully removed form public insurance scheme | | Policy driven  Outcome focused | | | Evidence of ineffectiveness  Budget restrictions | | | | Restriction | | | Reduced spending on service | | | | Reduced access  Denial of potentially beneficial care | | | Physical therapy (Gordon et al., 2007)  Routine eye exams (Kiran et al, 2013)  (Canada) | | | | (Gordon et al., 2007; Kiran et al., 2013; Landry et al., 2006) | | | | |  |
| Services that were defined, as insured but are no longer covered. | | Political process  Cost based | | | Adoption of new technology  Budget restrictions | | | | Removal | | | Improved efficiency of resource allocation | | | | Reduced access  Denial of potentially beneficial care | | | Various medical services  (Canada) | | | | (Elshaug, Watt, et al., 2009) | | | | |  |
| Full or partial removal of a service from a publicly funded insurance list. | | Evidence based process | | | Budget restrictions | | | | Reduction or restriction | | | Improved efficiency | | | | Reduced access  Denial of potentially beneficial care | | | Physical therapy  (Canada) | | | | (Landry et al., 2006) | | | | |  |
