# Supplementary material for: Conceptualising characteristics of resources withdrawal from medical services: a systematic qualitative synthesis
Source: Health Res Policy Syst. 2020 Oct 28;18:123. doi: 10.1186/s12961-020-00630-9 (PMC7592573; doi:10.1186/s12961-020-00630-9)
Supplement: Supplementary file 2 — Additional file 2: Appendix 3. References for the Fig. 1: Characteristics of resource withdrawal. [file 12961_2020_630_MOESM2_ESM.docx]

Appendix 3: References for the figure 1: Characteristics of resource withdrawal

1. Gallego G, Haas M, Hall J, Viney R. Reducing the use of ineffective health care interventions: an evidence check rapid review. 2010;(January):30.

2. Ibargoyen-Roteta N, Gutiérrez-Ibarluzea I, Asua J. Guiding the process of health technology disinvestment. Health Policy. 2010;98(2-3):218-226. doi:10.1016/j.healthpol.2010.06.018.

3. Pearson S, Littlejohns P. Reallocating resources: how should the National Institute for Health and Clinical Excellence guide disinvestment efforts in the National Health Service? J Health Serv Res Policy. 2007;12(3):160-165. doi:10.1258/135581907781542987.

4. National Health Committee. Think Piece: Towards a Plan for Better Targetting of Existing Health Services. Wellington; 2012.

5. Daniels T, Williams I, Robinson S, Spence K. Tackling disinvestment in health care services: The views of resource allocators in the English NHS. J Health Organ Manag. 2013;27(6):762-780. doi:10.1108/JHOM-11-2012-0225.

6. Joshi N, Stahnisch F, Noseworthy T. Reassessment of Health Technologies: Obsolescence and Waste. Ottawa; 2009.

7. Moreira T. Health care rationing in an age of uncertainty: a conceptual model. Soc Sci Med. 2011;72(8):1333-1341. doi:10.1016/j.socscimed.2011.02.026.

8. Carlsson P. Priority setting in health care: Swedish efforts and experiences. Scand J Public Health. 2010;38(6):561-564. doi:10.1177/1403494810377406.

9. Syrett K. A Technocratic Fix to the “Legitimacy Problem ”? The Blair Government and Health Care. J Health Polit Policy Law. 2003;28(4).

10. Fox DM, Leichter HM. Rationing care in Oregon: the new accountability. Health Aff. 1991;10(2):7-27. doi:10.1377/hlthaff.10.2.7.

11. Rosenthal MB, Newhouse JP. Managed care and efficient rationing. J Health Care Finance. 2002;28(4):1-10.

12. Giacomini M. The which-hunt: assembling health technologies for assessment and rationing. J Health Polit Policy Law. 1999;24(4):715-758. http://jhppl.dukejournals.org/content/24/4/715.short. Accessed January 6, 2015.

13. Leggett L, Noseworthy TW, Zarrabi M, Lorenzetti D, Sutherland LR, Clement FM. Health technology reassessment of non-drug technologies: current practices. Int J Technol Assess Health Care. 2012;28(3):220-227. doi:10.1017/S0266462312000438.

14. Mackean G, Noseworthy TW, Elshaug AG, et al. Health technology reassessment: the art of the possible. Int J Technol Assess Health Care. 2013;29(4):418-423. doi:10.1017/S0266462313000494.

15. Banta HD, Thacker SB. The case for reassessment of health care technology. Once is not enough. JAMA. 1990;264(2):235-240.

16. Robert G, Harlock J, Williams I. Disentangling rhetoric and reality: an international Delphi study of factors and processes that facilitate the successful implementation of decisions to decommission healthcare services. Implement Sci. 2014;9(1):123. doi:10.1186/s13012-014-0123-y.

17. Elshaug AG, Watt AMAM, Moss JRJJR, Hiller JJE. Policy Perspectives on the Obsolescence of Health Technologies in Canada. Ottawa; 2009.

18. Prasad V, Ioannidis JP. Evidence-based de-implementation for contradicted, unproven, and aspiring healthcare practices. Implement Sci. 2014;9:1. doi:10.1186/1748-5908-9-1.

19. Gordon M, Waines B, Englehart J, et al. The Consequences of Delisting Publicly Funded, Community-Based Physical Therapy Services in Ontario: A Health Policy Analysis. Physiother Canada. 2007;59(01):58. doi:10.2310/6640.2007.00002.

20. Kiran T, Kopp A, Moineddin R, et al. Unintended consequences of delisting routine eye exams on retinopathy screening for people with diabetes in Ontario, Canada. CMAJ. 2013;185(3):E167-E173. doi:10.1503/cmaj.120862.

21. Landry MD, Deber RB, Jaglal S, et al. Assessing the consequences of delisting publicly funded community-based physical therapy on self-reported health in Ontario , Canada : a prospective cohort study. 2005:303-307.

22. Elshaug AG, Moss JR, Littlejohns P, Karnon J, Merlin TL, Hiller JE. Identifying existing health care services that do not provide value for money. Med J Aust. 2009;190(5):269-273.

23. Elshaug AG, Hiller JE, Moss JR. Exploring policy-makers’ perspectives on disinvestment from ineffective healthcare practices. Int J Technol Assess Health Care. 2008;24(1):1-9. doi:10.1017/S0266462307080014.

24. Elshaug AG, Hiller JE, Tunis SR, Moss JR. Challenges in Australian policy processes for disinvestment from existing, ineffective health care practices. Aust New Zealand Health Policy. 2007;4:23. doi:10.1186/1743-8462-4-23.

25. Garner S, Littlejohns P. Do NICE’s recommendations for disinvestment add up? BMJ. 2011;343(August):349-351. Available from Highwire Press in http://link.worldcat.org/?rft.institution_id=129699&spage=349&pkgName=bmj&issn=0959-8154&linkclass=to_article&jKey=www.bmj.com&issue=7819&provider=highwire&date=2011&aulast=Garner,+Sarah&atitle=Do+NICE’s+recommendations+fo.

26. Gerdvilaite J, Nachtnebel A. Disinvestment: Overview of Disinvestment Experiences and Challenges in Selected Countries.; 2011.

27. Rumbold G, Allen K, Harris C. Disinvestment of Technologies and Clinical Practices in Health Services: Conceptual and Policy Perspectives. Melbourne; 2008.

28. Watt AM, Willis CD, Hodgetts K, Elshaug AG, Hiller JE. Engaging clinicians in evidence-based disinvestment: role and perceptions of evidence. Int J Technol Assess Health Care. 2012;28(3):211-219. doi:10.1017/S0266462312000402.

29. Haas M, Hall J, Viney R, Gallego G. Breaking up is hard to do: why disinvestment in medical technology is harder than investment. Aust Health Rev. 2012;36(2):148-152. doi:10.1071/AH11032.

30. Russell J, Greenhalgh T. Being “rational” and being “human”: How National Health Service rationing decisions are constructed as rational by resource allocation panels. Health (London). 2013;18(5):441-457. doi:10.1177/1363459313507586.

31. Donaldson C, Bate a, Mitton C, Dionne F, Ruta D. Rational disinvestment. QJM. 2010;103(10):801-807. doi:10.1093/qjmed/hcq086.

32. García-Armesto S, Campillo-Artero C, Bernal-Delgado E. Disinvestment in the age of cost-cutting sound and fury. Tools for the Spanish National Health System. Health Policy. 2013;110(2-3):180-185. doi:10.1016/j.healthpol.2013.01.007.

33. Karnon J, Carlton J, Czoski-Murray C, Smith K. Informing disinvestment through cost-effectiveness modelling: Is lack of data a surmountable barrier? Appl Health Econ Health Policy. 2009;7(1):1-9. doi:10.2165/00148365-200907010-00001.

34. Frønsdal KB, Facey K, Klemp M, Norderhaug IN, Mørland B, Røttingen J-A. Health technology assessment to optimize health technology utilization: using implementation initiatives and monitoring processes. Int J Technol Assess Health Care. 2010;26(3):309-316. doi:10.1017/S0266462310000309.

35. Martin E. Rationing in Healthcare.; 2015. http://ahha.asn.au/system/files/docs/publications/deeble_issues_brief_no_8_martin_e_rationing_in_healthcare.pdf.

36. Aaron H, Schwartz W. Rationing health care: the choice before us. Science (80- ). 1990;247(4941):418-422. doi:10.1126/science.2300804.

37. Schmidt D. The development of a disinvestment framework to guide resource allocation decisions in health services delivery organizations. 2010;(October).

38. Nuti S, Vainieri M, Bonini A. Disinvestment for re-allocation: a process to identify priorities in healthcare. Health Policy. 2010;95(2-3):137-143. doi:10.1016/j.healthpol.2009.11.011.

39. Haines T, O’Brien L, McDermott F, et al. A novel research design can aid disinvestment from existing health technologies with uncertain effectiveness, cost-effectiveness, and/or safety. J Clin Epidemiol. 2014;67(2):144-151. doi:10.1016/j.jclinepi.2013.08.014.

40. Hodgetts K, Hiller JE, Street JM, et al. Disinvestment policy and the public funding of assisted reproductive technologies: outcomes of deliberative engagements with three key stakeholder groups. BMC Health Serv Res. 2014;14(1):204. doi:10.1186/1472-6963-14-204.

41. Robinson S, Glasby J, Allen K. “It ain”t what you do it’s the way that you do it': lessons for health care from decommissioning of older people's services. Health Soc Care Community. 2013;21(6):614-622. doi:10.1111/hsc.12046.

42. Mitton C, Dionne F, Damji R, Campbell D, Bryan S. Difficult decisions in times of constraint: criteria based resource allocation in the Vancouver Coastal Health Authority. BMC Health Serv Res. 2011;11(1):169. doi:10.1186/1472-6963-11-169.

43. Russell J, Swinglehurst D, Greenhalgh T. “Cosmetic boob jobs” or evidence-based breast surgery: an interpretive policy analysis of the rationing of “low value” treatments in the English National Health Service. BMC Health Serv Res. 2014;14(1):413. doi:10.1186/1472-6963-14-413.

44. Bevan G, Brown LD. The political economy of rationing health care in England and the US: the “accidental logics” of political settlements. Health Econ Policy Law. 2014;9(3):1-22. doi:10.1017/S1744133114000127.

45. Meadowcroft J. Patients, politics, and power: government failure and the politicization of U.K. health care. J Med Philos. 2008;33(5):427-444. doi:10.1093/jmp/jhn022.

46. Mechanic D. Muddling through elegantly: finding the proper balance in rationing. Health Aff. 1997;16(5):83-92. doi:10.1377/hlthaff.16.5.83.

47. Mechanic D. Dilemmas in rationing health care services: the case for implicit rationing. BMJ. 1995;310(6995):1655-1659. doi:10.1136/bmj.310.6995.1655.

48. Gravelle H, Siciliani L. Ramsey waits: Allocating public health service resources when there is rationing by waiting. J Health Econ. 2008;27(5):1143-1154. doi:10.1016/j.jhealeco.2008.03.004.

49. Maxwell RJ. Why rationing is on the agenda. 1995;51(4):761-768.

50. Schwartz W, Mendelson DN. Eliminating waste and inefficiency can do little to contain costs. Health Aff. 1994;13(1):224-238. doi:10.1377/hlthaff.13.1.224.

51. Forbes A, Griffiths P. Methodological strategies for the identification and synthesis of “evidence” to support decision-making in relation to complex healthcare systems and practices. Nurs Inq. 2002;9(3):141-155. doi:10.1046/j.1440-1800.2002.00146.x.

52. Mullen PM. Is it Necessary to Ration Health Care? Public Money Manag. 1998;18(1):52-58. doi:10.1111/1467-9302.00104.

53. Redmayne S, Klein R. Rationing in practice : the case ofin vitro fertilisation. BMJ Br Med J. 1993;306(June):1521-1524.

54. Campbell A V. Defining Core Health Services: the New Zealand Experience. Bioethics. 1995;9(3):252-258. doi:10.1111/j.1467-8519.1995.tb00359.x.

55. Malone N, Rycroft-Malone J. Equity and rationing in the NHS: past to present. J Nurs Manag. 1998;6(6):325-332. doi:10.1046/j.1365-2834.1998.00086.x.

56. Hope T, Hicks N, Reynolds DJ, Crisp R, Griffiths S. Rationing and the health authority. BMJ. 1998;317(7165):1067-1069.

57. Plomer A, Smith I, Martin-Clement N. Rationing policies on acces to in vitro fertilisation in the national health service. Reprod Health Matters. 1999;7(14):60-70.

58. Klein R, Maybin J. Thinking about Rationing.; 2012.

59. Giacomini M, Hurley J, Stoddart G. The many meanings of deinsuring a health service: the case of in vitro fertilization in Ontario. Soc Sci Med. 2000;50(10):1485-1500. doi:10.1016/S0277-9536(99)00394-9.

60. Landry MD, Deber RB, Jaglal S, et al. Assessing the consequences of delisting publicly funded community-based physical therapy on self-reported health in Ontario, Canada: a prospective cohort study. Int J Rehabil Res. 2006;29(4):303-307. doi:10.1097/MRR.0b013e328010badc.

61. Elshaug AG, Watt A, Mundy L, Willis C. Over 150 potentially low-value health care practices: an Australian study. Med J Aust. 2012;197(November):556-560. doi:10.5694/mja12.11083.

62. Polisena J, Clifford T, Elshaug AG, Mitton C, Russell E, Skidmore B. Case Studies That Illustrate Disinvestment and Resource Allocation Decision-Making Processes in Health Care: a Systematic Review. Int J Technol Assess Health Care. 2013;29(02):174-184. doi:10.1017/S0266462313000068.

63. Watt AM, Elshaug AG, Willis CD, Hiller JE. Assisted reproductive technologies: a systematic review of safety and effectiveness to inform disinvestment policy. Health Policy. 2011;102(2-3):200-213. doi:10.1016/j.healthpol.2011.07.007.

64. Mitton C, Levy A, Gorsky D, MacNeil C, Dionne F, Marrie T. Allocating limited resources in a time of fiscal constraints: a priority setting case study from Dalhousie University Faculty of Medicine. Acad Med. 2013;88(7):939-945. doi:10.1097/ACM.0b013e318294fb7e.

65. Griffiths S. Prioritizing care in a resource-limited health service. Curr Paediatr. 2002;0.

66. Klein R, Day P, Redmayne S. Rationing in the NHS: The dance of the seven veils in reverse. Br Med Bull. 1995;51(4):769-780.

67. Klein R. Priorities and rationing: pragmatism or principles. BMJ Br Med J. 1995;(September).

68. Klein R. Can we restrict the health care menu? Health Policy (New York). 1994;27(2):103-112. doi:10.1016/0168-8510(94)90075-2.

69. Schwartz W, Mendelson DN. Why managed care cannot contain hospital costs--without rationing. Health Aff. 1992;11(2):100-107. doi:10.1377/hlthaff.11.2.100.

70. Jacobs N, Schneider H, Van Rensburg HCJ. Rationing access to public-sector antiretroviral treatment during scale-up in South Africa: implications for equity. African J AIDS Res. 2008;7(1):19-27. doi:10.2989/AJAR.2008.7.1.3.431.

71. Health Policy Advisory Committee on Technology. Disinvestment in Australia and New Zealand.; 2013.

72. Sibbald SL, Singer P a, Upshur R, Martin DK. Priority setting: what constitutes success? A conceptual framework for successful priority setting. BMC Health Serv Res. 2009;9:43. doi:10.1186/1472-6963-9-43.

73. Lock RJ. Rational requesting or rationing testing? J Clin Pathol. 2004;57(2):121-122. doi:10.1136/jcp.2003.11122.

74. Paprica A. Reassessing Existing Funded Health Services and Products to Support Appropriate Care.; 2013. cfhi-fcass.ca.

75. Gerdvilaite J, Nachtnebel A. Disinvestment. Overview of disinvestment experiences and challenges in selected countries (Structured abstract). Vienna Ludwig Boltzmann Inst fuer Heal Technol Assess. 2011:-.

76. Redmayne S, Klein R. Rationing in practice: the case of in vitro fertilisation. BMJ. 1993;306(June):1521-1524. doi:10.1136/bmj.306.6891.1521.

77. Prasad V, Cifu A, Ioannidis J. Reversals of established medical practices: Evidence to abandon ship. JAMA J Am Med Assoc. 2012;307(1):37. doi:10.1001/jama.2011.1960.

78. Robinson S, Williams I, Dickinson H, Freeman T, Rumbold B. Priority-setting and rationing in healthcare: evidence from the English experience. Soc Sci Med. 2012;75(12):2386-2393. doi:10.1016/j.socscimed.2012.09.014.

79. Garner S, Littlejohns P. Disinvestment from low value clinical interventions: NICEly done? BMJ. 2011;343:d4519. doi:10.1136/bmj.d4519.
